# Hospital and Institutional News

**Published:** 1920-03-06

**Authors:** 


### HOSPITAL AND INSTITUTIONAL NEWS.

### SIR WILLIAM OSLER'S SUCCESSOR.

It is understood that the name of Sir A. E. Garrod, who was recently appointed Director of the Clinical Unit at St. Bartholomew's Hospital, has been put forward as the probable Regius Professor of Medicine at Oxford University in succession to the late Sir William Osler. Sir A. E. Garrod is sixty-two years of age, and besides holding his position on the staff at St. Bartholomew's, is also Consulting Physician to the Hospital for Sick Children, Great Ormond Street. During the war he served in the Mediterranean areas and was twice mentioned in dispatches, being made K.C.M.G. in 1918.

### A MEMORIAL.

A public meeting has been called in the lecture theatre of the museum at Oxford on Saturday March 6 at 3.30 p.m., to consider what steps should be taken to perpetuate, in some appropriate manner, the memory of Sir William Osler. All immediately concerned with the teaching of medicine at Oxford have been invited, and many university officials and heads of houses have intimated their intention of being present. Notification of such intention should be sent to the Interim-Secretary, Professor Gunn, at the Museum, Oxford.

### TO ENCOURAGE RESEARCH.

On Tuesday last a deputation from the Medical Committee of the House of Commons waited on Mr. Balfour and asked that the Government should initiate a system of awards for medical and scientific discoveries which, it was proposed, should be distributed on the lines of the Nobel prizes. The moderate annual sum estimated to be sufficient for the object in view is £20,000, and it is hoped that the Treasury will not raise any serious opposition to this wise investment of money. Men capable of scientific discovery are very scarce, and when one is found he should be encouraged and helped; the chances being that he will repeat his good work. How, exactly, it is proposed that the money, if granted, would be distributed is not disclosed. It is said that the medical members of Parliament do not favour a further endowment of the Medical Research Committee. On this point the medical correspondent of *The Times* makes the curious reflection that "This Committee has done splendid work, but it would be a difficult matter, nevertheless, to guarantee that original genius will not show itself outside the ranks of its staff."

### SPECIAL NEUROLOGICAL BOARDS.

An Army Council instruction recently issued states that the arrangements made in March 1918 regarding medical boards on neurasthenic officers are cancelled, and that there will now be special Ministry of Pensions' Boards designated "Special Neurological Boards." No officer suffering from any of the diseases of this type will be marked permanently unfit for service unless one member of the board has special knowledge of diseases of the nervous system and concurs in the finding. An officer certified by a medical board (including a specialist) to be unfit will usually remain in hospital or with his unit pending instruction from War Office as to his disposal, but if there is no medical objection and no further active treatment is required he may be allowed to proceed to his home. These instructions also apply to nurses.

### INDIAN MEDICAL SERVICE.

The Secretary of State for India makes the following announcement: Fully qualified European medical practitioners are required for temporary service with Indian troops on special rates of pay. Applicants must be British subjects of European descent and should not be more than 35 years of age. If accepted for appointment they will be required to enter into a contract to serve for two years. The rates of pay will be Rs. 700 per month for a lieutenant, Rs.750 per month for a captain. Three or more years' service as a medical officer in the home, Colonial, or Indian forces is necessary to qualify a candidate for the higher rate. Officers will be provided with free passages both ways. Wives and families will be sent out at Government expense in the autumn of 1920, provided that shipping is available, and will be given free return passages. An outfit allowance of £50 will be granted except in the case of officers who have served under the War Office or the Government of India, to whom £20 only will be issued. Further particulars and forms of application may be obtained from the Secretary, Military Department, Room 157, India Office, Whitehall, S.W.1. Envelopes should be clearly marked in the top left-hand corner "Temporary I.M.S."

### SIR HENRY BURDETT, K.C.B.

Sir Henry Burdett, who is suffering from the effects of severe and protracted overwork, has been compelled to take a complete rest. He is forbidden by his doctors to transact business or to do work of any kind.

### THE NEW SECRETARY OF THE METROPOLITAN.

If the freshness of youth is an important factor in success the new Secretary of the Metropolitan Hospital, Mr. Guy B. Dale, who is only twenty-seven, should do well. He comes from the Secretaryship of the Beckett Hospital, Barnsley, and was previously Assistant Secretary of the North Staffordshire Infirmary, Stoke-on-Trent. Mr. Dale has a good record of war service. He enlisted in December 1915, and served with the Royal Garrison Artillery as Signal Corporal, with two years of this service in France. He has been awarded the Military Medal with Bar. The Beckett Hospital is appealing at present for £20,000, so Mr. Dale will have had recent experience in money-raising which will serve him in good stead at the Metropolitan.

### A LEAD FROM NOTTINGHAM.

At Nottingham the Monthly Board of the General Hospital reports good news in connection with the appeal for an additional £20,000 a year. The employees of one local firm have promised to subscribe £1,000 a year, in recognition of which the employers have raised their annual subscription from 50 guineas to £500.

### MODERN METHODS OF APPEAL.

The power of big schemes of publicity—which was one of the lessons learnt from successful war-time propaganda in connection with recruiting, war savings, and so forth—is increasingly recognised. The latest instance is to be found in the full front-page announcement in the *Nottingham Guardian* on Friday, February 27, in connection with the Nottingham General Hospital, on the day before "Hospital Saturday." A bold "streamer" (to use newspaper parlance) ran across the top of the page, and a double-column heading—"Critical Position: More Income or Fewer Beds"—focussed attention. The text consisted of an expression of thanks to those who have responded to the appeal for an additional £20,000 this year, summarised details of some of the more notable offers of help already made, and the plain statement that the hospital's debt is increasing and the bank overdraft is £28,000. The Monthly Board has reluctantly come to the conclusion that unless the promises received before April show reasonable prospect that the income will meet the expenditure, it will be compelled to recommend that a number of beds be closed. "The decision rests with the public, not with the Monthly Board." There must either be more income or fewer beds. These facts are admirably set forth, and the appeal ends with a long list of subscribers. These are in sections showing the extent to which employees and employers respectively have promised to increase their subscriptions; new subscribers and donors since January 1919; and, finally, those employers who have promised to base their subscriptions on the amounts given by their workpeople. It is an excellent piece of publicity. The measure of its success will be some indication of the value of publicity itself. However great that value it is necessarily limited. In the end the voluntary hospital has to rely upon the belief of the public in the principle of personal service for which it stands, and, should that belief decay, no amount of publicity can replace the driving force of this primary sense of public spirit.

### AN INVISIBLE CROWD AT CARDIFF.

If 223 people can give £100,000, and if half that sum has been subscribed in donations of four figures and over, how many people are required to raised £50,000 more on the assumption that the biggest donors have already come forward? That is the problem before Cardiff citizens to-day, where of the £150,000 asked for on behalf of King Edward VII. Hospital, £100,000 has been very quickly raised. An analysis of the donations makes interesting reading. The largest donor gave £25,000. Including him, thirty-six persons have given over £1,000 each. The total subscribed at the moment of writing is £100,754, and to this we understand that only 223 people have contributed. This number, moreover, includes several donors of one pound or less; a donor of 2s. 9d. is honourably recorded. This contrast between the total subscribed and the handful of persons who have combined to subscribe it is very striking. But the inference has not been driven home. It is, frankly, this. No hospital appeal in Cardiff, no matter what sum is raised, can be regarded with pride by the city if the appeal, in spite of the widest publicity, succeeds in interesting only two or three hundred people. The apathy of persons in Cardiff must be appalling (there is no other word), if only so few are moved by the great campaign in their midst. For if the figures be accepted for the moment at their face value merely, we are forced to this conclusion that the only class which (as a class) has given anything in proportion to its numbers is the class of members which can give about £100 apiece or more. That class must necessarily be small compared with other classes. For the one man who can give £100 to anything, there must be at least twenty who can give £5, at least one hundred who can give £1, at least two hundred who can give 10s., at least four hundred who can give 5s. Where are these people hiding their heads? The hospital serves half a million of them or thereabouts, so that it cannot be pretended that we have invented their existence. In fact, 499,777 persons in the Cardiff area have given nothing at all. It would be better to insist on this number than on the handful which has helped. Many more people will be interested in the larger figure, and, we hope, ashamed to be numbered therein.

### MORE DAYLIGHT.

We are asked, in view of the fact that the new Daylight Saving Bill will shortly be considered in the House of Commons, to draw attention to the advantages of daylight saving as already demonstrated in this and other countries, and to put forward to the hospital world the proposal that Willett's original scheme may be more closely followed, by advancing the clock yet *another* hour from the middle of May till the end of July. This would certainly mean that all who are engaged in work would get an extra hour of daylight in the evening for recreation, much of which time would unquestionably be spent in allotment and other productive home work. For several weeks it would give us daylight up to 10.30 or even 11 o'clock, and even the very early riser would not be inconvenienced, as it would be light by five during the same period. On the score of economy, too, the case for the extra hour is even stronger and the suggestion is one which should most certainly receive due consideration.

### THE COST OF PETROL.

The increased price of motor spirit and its tendency constantly to rise will undoubtedly affect every section of the community and not the least that constituted by the medical and hospital world.

The situation calls for strong and immediate action on the part of the Government. As a means to this end, when the situation may be brought to one satisfactory understanding, whatever the immediate effect on the price of petrol may be, the petition to be presented to the Premier by the Automobile Association is likely to achieve a most valuable result. Therefore would we urge that every possible signature be obtained on the forms the A.A. is circulating, no matter in what profession, trade, or occupation the persons may be interested. Any motoring non-members or others who will give the Association their support in this national work are asked to send their names and addresses to The Secretary, Fanum House, Whitcomb Street, London, W.C. 2, when petition forms will be forwarded to them. This is not a matter for the motorist alone, but for every member of the community. Everyone must suffer directly or indirectly as the price of motor fuel reaches more and more preposterous figures.

### HIGH PRICES AND STREET COLLECTIONS.

The tentacles of high prices are at last successfully attacking the small articles sold in connection with street collections, and we learn that on Alexandra Day, the ninth celebration of which will be held on June 23, a paper rose will be sold as the penny emblem, because cotton has risen to such a price as to make the rose of that material prohibitive. It is hoped that the day will be kept throughout the London area, and between three and four hundred places have already announced their intention of joining the movement in the provinces. Roses are being sent to Canada, and many of the steamship companies have promised their help. Last year a sum of nearly £600 was received from various celebrations on Alexandra Day on ships in all parts of the world. It should be made clear that the proceeds of the collection go to other charities besides hospitals. As in most street collections, the casual giver has but a hazy idea as to what becomes of his money.

### THE NATION'S HEALTH.

The contents of the recent report by the Ministry of National Service on the state of the nation's health will not in its main findings come as a surprise to readers of this Journal, but although we expected that alarming figures would be produced, it is disappointing to find some of them as black as they are. Of the men of military age examined between November 1917 and October 1918 only one in three was up to the normal standard of health and strength, and one in ten was totally and permanently unfit for any form of military service. True it is that this occurred in a period relatively late in the war, but the facts bring before us a situation which must be considered seriously. One pleasing conclusion appears, however—namely, that our population increases in spite of a decreasing birth-rate, and this due to a general prolongation of life. There is a greater average age for population, and this is apparently accompanied by greater physical efficiency in the more advanced years of life than has been general in previous generations.

### REFORMS AT THE ROYAL PORTSMOUTH HOSPITAL: INCREASED REPRESENTATION OF LABOUR.

For the purpose of reconstituting the committee of management of the Royal Portsmouth Hospital, a special meeting of the subscribers was held a fortnight ago at the Town Hall. Mr. Woolmer White, J.P., C.C., presided, and the resolutions, which were submitted by Sir Harold Pink and seconded by Mr. T. H. F. Lapthorn, were carried. The object of these resolutions was to secure for the working classes a larger share than they have held hitherto in the management of the hospital. Under the new rules the committee of management will be so constituted that one half of the members shall consist of three of the vice-presidents, the treasurer, the chaplain, the honorary architect for the time being, three members of the honorary senior medical and surgical staff, two members of the honorary assistant medical and surgical staff, one member of the honorary dental staff, and of so many additional members to be elected by the annual subscribers as, with those above mentioned, will make up one-half of the total membership of the committee. The other half of the committee is to be constituted as follows:—Fourteen members to be nominated by the undermentioned bodies while subscribing to the hospital funds: one by the Ladies Hospital Linen League, two by the Royal Naval Barracks Canteen Committee, one by the R.M.A. Canteen Committee, one by the R.M.L.I. Canteen Committee, three by the Portsmouth Trades and Labour Council, four by the Workmen's Committee, H.M. Dockyard, two by the Portsmouth Friendly Societies' Council. (These fourteen nominated members will hold office for three years, but will be eligible for re-election). Ten members to be nominated annually by the undermentioned bodies while subscribing to the hospital funds:—one by the Portsmouth Corporation, one by the Board of Guardians, two by the Portsmouth Hospital Sunday Fund, two by the Portsmouth Hospital Saturday Fund, one by the Gosport and Alverstoke Local Committee, one by the Fareham and District Hospital Saturday Fund, and two by the Portsmouth Teachers' Association. These new rules are the natural corollary to the splendid response which has been made by the working classes to the recent appeals of the hospital for increased financial support.

### A HOPEFUL CHANGE AT PORTSMOUTH.

Since Sir Harold Pink became actively interested in the management of the Royal Portsmouth Hospital, a healthy improvement has occurred, from whatever cause observers may care to assign. To increase local support, which has been notoriously halting in the past, and to rebut the charge of exclusiveness, the Committee has evolved a new constitution for itself. The plan, as Mr. T. H. F. Lapthorn neatly put it, is to divide the management between those who find the money and those who benefit in the wards. In this way, he argues, public confidence can be restored completely. The Committee has therefore decided that one-half of its members shall be nominated by subscribing organisations. The other half will consist of the usual office bearers, and representatives of the honorary medical staff. It is thus hoped to enlist the support of the Workmen's Committee of H.M. Dockyard and Kindred Bodies, and we hope that the increased representation now given will lead to gifts no less generous on the part of the men. For if the latter are to demand representation in proportion to their numbers, the hospital is entitled to expect support no less generous than the representation given. Indeed, the hospital should expect much more. Bargains are no part of the voluntary spirit. A decent man gives to hospitals to benefit the sick, not to purchase representation for himself. Thus, as always, every voluntary hospital reposes on the principle of personal service, and where that gospel is not preached, or when it is not practised, no financial scripts or commercial methods of bargaining can save it eventually from decay. Such methods are alien to its spirit, which must express itself *through* organisations, but not sacrifice itself to ways and means.

### A HALF-EMPTY HOSPITAL.

One hospital is complaining of a scarcity of patients. This is the Southport Convalescent Hospital, which, during the winter, seldom has its beds fully occupied. There are 360 beds, and of these about 100 only are in occupation during the winter. The cost per patient is said to be at present three guineas for three weeks; the charges, on the other hand, for this period are £2 14s. Subscribers are urged to keep the beds fully occupied in order to reduce the cost. It is believed that the beds are empty in winter because subscribers do not realise that the institution is properly heated and that "facilities for recuperation and entertainment" are maintained. The institution now possesses a woman governor, and the medical superintendent, Dr. Payne, has been complimented on his management of the men patients in enforcing the rules without occasioning any serious trouble. We hope that the employers in Lancashire industrial towns will see that the vacant beds are used by any of their men who need care after illness or accident.

### A CHURCH ACQUIRED BY A HOSPITAL.

The Greenwich Road Congregational Church has been acquired by the Miller General Hospital. The site will be used, first, to relieve the present congestion in the out-patient department, and, finally, for part of the general scheme of extension. As soon as the church is vacated the alterations will begin, and to hasten their completion Mrs. Spencer Stidolph, of Larydale, Greenwich, has promised £1,000 towards the cost. A good-bye service was held in the church on February 23, and the building will soon be used for medical ministration to out-patients. The hospital architect, who is concerned with the adaptation of buildings, has been set many curious problems before now, but there must have been few, if any, precedents for the adaptation of a church to institutional uses.

### A DAY'S PAY FOR THE HOSPITAL.

A big scheme of extension is planned for the Royal Infirmary, Preston, and the way has been prepared by a gift of £5,000 from Lord Ashton, who has been generous to the hospital on many occasions. The plan is to build a new children's hospital in the grounds of the infirmary, as part of the Present War Memorial. The sum of £50,000 is needed and the employers and the employees in the cotton, engineering, and building trades have met in frequent conference upon the matter. In each case it has been recommended that the workpeople should give a full day's pay, and that the employers should give a sum equal to the men's subscription. All the trades and businesses in Preston are coming into line with this agreement. By this means it is expected that £25,000 will be raised by the cotton mills alone, and the success of the scheme is virtually certain. We hope also that the plan may prove the beginning of a new scheme, and that Mr. John Gibson, the secretary, may be able to assure the maintenance of the new buildings by a permanent co-operation between employers and employed.

### A MUNICIPAL BARGAIN.

Several excellent points were made by speakers at the recent annual meeting of the Royal Devon and Exeter Hospital, and this shows that annual meetings are not dull by nature, but are made dull by the opportunities which the speakers miss. For example, Mr. Shirley Steele-Perkins devoted some critical attention to the recent powers of local authorities under the Health Acts. He reminded the audience that under the Child Welfare Act beds for children under five years old must be provided in hospitals. The Devon and Exeter Hospital has been adopted by the Exeter City Council under that Act, and to the hospital the Council is indebted for the provision of the beds required. But this provision has not yet led the City Council to increase its traditional subscription of £10 a year. On the other hand, as Mr. Donald Wilson aptly added, rates have not remained stationary. The hospital, he said, was paying to the Council about £300 a year for rate, and something like £50 a year for water. This is a good example of municipal bargaining, which, however, we have no doubt that the City Council will be prompt to revise once it has been shown what it owes to the hospital.

### THIS WEEK'S DRUG MARKET.

Several interesting price changes are to be noted. Manufacturers of iodides have advanced their prices; this is in consequence of increased working costs and not on account of any change in the position of crude iodine. Calomel and other salts of mercury have also been advanced in price in consequence of the higher value of quicksilver. Castor oil is dearer, as also are aloin, apiol, atropine, caffeine and strychnine. Potassium permanganate is still in short supply and the price tends upwards. Citric acid is scarcer and dearer. Several synthetic drugs have an upward price tendency, including aspirin, hexamine, antifebrin and phenazone. Ipecacuanha is again dearer.

